# Who enrols in voluntary micro health insurance schemes in low-resource settings? Experience from a rural area in Bangladesh

**DOI:** 10.1080/16549716.2018.1525039

**Published:** 2018-10-05

**Authors:** Shehrin Shaila Mahmood, Syed Manjoor Ahmed Hanifi, Mohammad Nahid Mia, Asiful Haidar Chowdhury, Mahabubur Rahman, Mohammad Iqbal, Abbas Bhuiya

**Affiliations:** a Universal Health Coverage, Health Systems and Population Studies Division, icddr,b, Dhaka, Bangladesh; b Centre for Global Health, Population and Policy, University of Portsmouth, Portsmouth, UK

**Keywords:** Health financing, enrolment, Outreville’s insurance demand framework, moral hazard, Bangladesh

## Abstract

**Background**: Micro health insurance (MHI) has proved to be a potential health-financing tool for many developing countries. Bangladesh also included MHI in its current health-financing strategy which aims to achieve universal health coverage. However, low uptake, low renewal and high dropouts have historically challenged financial sustainability of these schemes.

**Objective**: This study aims to identify factors influencing people from low-resource settings, particularly those from Bangladesh, to enrol in MHI schemes.

**Methods**: The study analyses the ‘*Amader Shasthya*’ MHI scheme operating in Chakaria, a sub-district under Cox’s Bazar district, Bangladesh. A household survey was carried out during May–June 2016 among 2,000 households from the scheme coverage area. The Outreville’s insurance-demand framework was used to identify enrolment influencing factors. Multivariate logistic regression analysis was carried out to identify significant influencing factors of enrolment.

**Results**: Enrolment influencing factors were identified in four dimensions: economic, socio-cultural, demographic and structural. Households with the main income earner having 10+ years of schooling (odds 1.9 [CI 1.2–2.9] compared to illiterate), having financial literacy (odds 1.5 [CI 1.2–1.8] compared to financially illiterate) and being a public/private service holder (odds 1.6 [CI 1.1–2.4] compared to menial labour) were more likely to enrol. Membership in development programmes of NGOs also influenced enrolment decision significantly (odds 1.3 [CI 1.0–1.5]). The presence of chronic illness in household encouraged enrolment (odds 1.5 [CI 1.2–1.8]). Households living closer to health centres were more likely to enrol (odds 2.1 [CI 1.6–2.7]) compared to those living further away.

**Conclusion**: The findings are expected to have significant implications in terms of designing similar health insurance schemes, particularly in terms of designing demand-driven and context adapted schemes that have greater potential to attract a larger client pool, ensure effective risk pooling and eventually expedite the achievement of universal health coverage in low-resource settings.

## Background

Micro Health Insurance (MHI), a customized health insurance scheme, is recently being used in the developing world to provide its citizens, particularly the poor and those involved in the informal sector, protection against the financial risk of ill-health. The potential of MHI in providing financial protection to the poor in low-income countries has been highlighted in earlier studies [1–3]. Bangladesh, in its current national health-financing strategy, has also identified a social health insurance mechanism to pave its way towards universal health coverage. The financing strategy also highlights the role of MHI or community health insurance to work as a transitional pathway towards establishing a social health insurance mechanism countrywide. Further, MHI can play a vital role in including clients from the informal sector who are mostly out-of-reach of the existing national revenue collection systems. Having said this, the concept of insuring against the risk of ill health is not very common in Bangladesh. Private health insurance has a meagre share of less than 1% in total health expenditure. Besides private health insurance, only a few attempts have been made by the NGOs to run micro health insurance schemes in various parts of the country. However, these organizations have struggled to introduce and sustain MHI schemes in Bangladesh to minimize the financial burden on people resulting from sudden health shock. Low level of uptake, high dropout rates and financial sustainability remain as major challenges in using MHI as an alternative health financing mechanism [4,5]. icddr,b, an international research organization also initiated an MHI scheme known as ‘*Amader Shathya*’ meaning ‘our health’ in 2012 in a rural area in Bangladesh. The scheme reached an enrolment rate of 20% by the end of the first three years of operation [6]. Although this rate is comparable to most schemes being operated in similar settings, it is still low when considering the financial sustainability of the scheme. The low level of enrolment into MHI schemes lead to the concern that, if only small numbers enrol, the economic or actuarial basis of the MHI scheme will be undermined threatening financial sustainability. Several studies have found that risk pooling remains limited for MHI schemes due to their small size [7]. On the other hand, when a sufficiently large number of people join a scheme, following the law of large numbers [8], projected claims can be estimated with a high degree of confidence. This allows the insurer to keep the premium more affordable for their clients, particularly for the poor, as now they do not have to include a large margin for error in their product pricing [9]. Therefore, a larger client pool is of particular significance for MHI schemes to ensure effective risk pooling and making healthcare affordable to all.

In this context, it is thus important to understand the determinants of enrolment in the MHI programmes. Although studies have been carried out in other developing countries to investigate the factors influencing enrolment into health insurance schemes [10–12], few have taken a holistic approach where the multidimensionality of demand for health insurance has been taken into account. Further, exploring people’s preference in a setting like Bangladesh, where the concept of health insurance is comparatively new is almost non-existent. The few studies carried out in Bangladesh have looked into the effect of MHI on various development indicators including health, poverty and the like [13,14]. The current study thus analyzes determinants and their effect on enrolment into the *‘Amader Shasthya’* MHI scheme in rural Bangladesh using the Outreville’s insurance demand framework considering four major dimensions of demand: Economic, social and cultural, demographic, and structural.

## Methods

The Outreville’s insurance demand framework [15] has been used to explore the determinants of enrolment in this study. The econometric model derived from the framework looks at the determinants from four different aspects: (a) Economic factors (e.g. wealth, occupation) (b) social and cultural factors (education, religion, financial literacy, etc.) (c) Demographic and health factors (age, sex, household size, marital status, health status etc.) (d) structural and other factors (NGO membership, distance between household and health centres, etc.)

## Study area

The study was carried out in Chakaria, an *Upazila* (sub-district) under the Cox’s Bazar district of Chittagong Division of Bangladesh. Typical of rural Bangladesh, Chakaria, is situated in the southeast part of the country and is home to a population of around 523,317 (2016 estimate) [16]. The major economic activities in the area include agriculture, forestry and sea fishing. Healthcare delivery in Chakaria comprises public, private and non-government organizations (NGO). At present, the Upazila Health Complex of the government and five private hospitals/clinics provide health-care services to the people of Chakaria. At the union level, 43 community clinics, 2 union sub-centres and 15 Union Health and Family Welfare Centres (UHFWCs) of the government provide health-care services [17]. Additionally, the MHI scheme operating in the area provides primary level out-patient healthcare through four village health posts (VHPs) which are run by icddr,b appointed paramedics five days a week and once a week by physicians. Village Health Posts arehealthcare centres setup by the community members in Chakariawith technical assistance from Chakaria Community Health Projectof ICDDR,B. Apart from these, the healthcare services of nearly 500 village doctors (allopathic and homoeopathic) dominate the health service delivery in this area. Like the rest of the country, the major share of healthcare expenses is borne out-of-pocket by the community members [18]. Public health services, although provided free of charge, are hampered by shortage of supplies (medicine etc.), crowding, ill behaved providers, and corruption in terms of unofficial payments [19].

Chakaria is one of the field sites of icddr,b and hosts a health and demographic surveillance system (HDSS).

## The micro health insurance scheme *‘Amader Shasthya’*

The MHI scheme in Chakaria is named ‘*Amader Shasthya*’ meaning ‘our health’. The scheme was initiated in February 2012. Awareness raising and promotional campaign about MHI was carried out in 4 of the 19 unions of Chakaria where there were 16,852 households. A five member marketing team carried out awareness raising and sensitization among community people about features and benefit packages of Micro Health Insurance Scheme. The scheme went through a number of modifications based on learning at various stage of operation [20]. The scheme enrols households against a yearly premium and in exchange it entitles the members of the enrolled family to get free outdoor services at the Village Health Posts (VHP) in Chakaria run by paramedics and two MBBS doctors; inpatient care in designated hospitals; discounted drug and diagnostic cost and other related hospitalization costs up to a yearly limit. The scheme currently offers two packages to cater to the needs of different groups within the community: Indoor and Outdoor [20]. Evidence from earlier studies revealed 20% households (a total of 5021 out of 25,513 households) in the catchment area of MHI ever enrolled in the scheme during first three years of implementation. Both enrolment and renewal have shown an increasing trend over time [21].

Against a yearly premium of BDT 1,200, the Indoor package provides consultations by MBBS doctors and trained Sub-Assistant Community Medical Officers (SACMO) at the VHPs and hospitalization services at a partner hospital (Zamzam Hospital) in Chakaria. The hospitalization services include hospital admission, general bed charge, and discount on drugs, pathology, X-ray, ultrasound and operations. Under this package, the yearly ceiling per household is BDT 54,000 and per household member is BDT 9,000. The outdoor package, on the other hand, provides member households with consultations from MBBS doctors and SACMO based at VHPs located in the villages of Chakaria, 20% discount on prescribed drugs and discounted services at partner diagnostic centres in exchange of a yearly premium of BDT 500. For this package, the household has a maximum yearly ceiling of BDT 30,000 and for individuals the yearly ceiling is BDT 5,000. In addition, to support the below poverty line (BPL) population (the bottom 20% of the population) the scheme introduced a poor card at a subsidized rate of 200 BDT per household per year. With this package the poor can avail all the entitlements of an outdoor package. Membership in each package expires after one year and is expected to be renewed for the next one year. The scheme in presented in Table 1

**Table 1.** The ‘*Amader Shasthya’* scheme.

Package type	Premium/year/household (1USD = BDT 83.7)	Benefit	Ceiling
Inpatient	BDT 1200	Consultation by MBBS doctors and trained medical assistants 20% discount on drug from partner pharmacy Hospitalization services: hospital admission, general bed, drug, pathology, X-ray, ultrasound, operation Discount on OPD service from partner hospital	BDT 54,000/household/year BDT 9,000/individual/year
Outpatient	BDT 500	Consultation by MBBS doctors and trained medical assistants 20% discount on drug from partner pharmacy 20% discount on diagnostic services from partner facility Discount on services from partner hospital	BDT 30,000/household/year BDT 5,000/individual/year
Special outpatient	BDT 200		

## Study design and data

Data for the current study came from a community survey carried out in Chakaria during May–June 2016 approximately 4 years after the MHI commenced. Following a case-control study design a total of 2,000 households from the 54 villages of the 4 unions where *‘Amader Shasthya’* operates were included in the survey. Among these 2000 households, 1000 were randomly selected from the 6,221 households who ever enrolled into the MHI scheme to serve as cases. To ensure representation of households from all the three packages, these 1000 households were then proportionately selected according to the share of the three package members in the total pool. 150 households were from inpatient package, 600 households from outpatient package and 250 households from the special outpatient package. Another 1000 households were randomly selected from non-member households of the same area to serve as control households. Of the 2000 respondents, 44 could not be interviewed for their non-availability; 34 were absent at the time of the visit, 8 migrated out of the area and 2 respondents refused to be interviewed. Finally, 1956 individuals aged 18 years or older were successfully interviewed. Information was collected on: socioeconomic and demographic characteristics of household members including the main income earner, health-seeking behaviour for out-patient, in-patient care, treatment of chronic disease, and, out-of-pocket expenses for different categories of healthcare.

Following completion of a five-day training, 22 local female interviewers with at least 10 years of schooling formed the data collection team. Two supervisors supervised the data collection process. Data were collected through open data kit (ODK) collect version 1.4.7 using android-based tablet phone. All the filled-up questionnaires were checked twice for completeness and for any inconsistencies before the data were sent to the server database. The supervisors revisited 2% of the cases to check data consistency and any anomalies found were then sorted out in consultation with the data collector.

The online HIS for the programme stores information on member profile (i.e. information on all household members who are members of the scheme) as well as date of joining the scheme, package utilization, treatment received, claim settlement and reimbursement, and healthcare costs. Whenever a member visits one of the health centres (VHPs), the healthcare provider stores his/her treatment details in the online database. Members are informed during registration to the scheme about possible use of data acquired through this online HIS for research activities. Written informed consent has been taken from the members regarding use of information. The data acquired through this online HIS have been used in the analysis.

Ethical Review Committee of icddr,b provided approval for the current research. Informed written consent was taken from all interviewees regarding publication of study findings, and confidentiality and anonymity of respondents were ensured.

## Definition of variables

In search of factors that influence uptake of health insurance scheme the dependent variable of concern in our current study ‘Enrolment’ was defined as a binary choice variable where members hold a value of ‘1ʹ and non members ‘0ʹ. Among the independent variables ‘Religion’ (Islam = 1, other = 0), ‘Marital status’ (ever married = 1, never married = 0), and ‘Age’ (< 30 = 1, ≥ 30 = 0) were coded as binary variables. The variable ‘Education’ recorded years of completed education in school. It was a categorical variable with four categories (none, 1–5 years of schooling, 6–10 years of schooling, and 10+ years of schooling). ‘Occupation’ was a categorical variable with five categories (day-labour, farmer, private/public service holder, businessman and others). ‘Household socioeconomic status’ was defined using asset index. Asset information of all study participants was collected which was then used to calculate asset index. Based on the asset index each household was classified into quintiles where the first quintile consists of the poorest 20% of the households and the fifth quintile consists of the wealthiest 20% of the households [22–24]. ‘Household size’ was a categorical variable with three categories (1–3 members, 4–6 members and 7+ members) indicating the total number of household members in a household. ‘Financial literacy’ was determined based on the main earner’s ability to answer four key questions which were designed to test their knowledge and understanding of the concept of financial transaction, loss, savings and interest. A financial literacy index was calculated based on number of correct answers. The index ranged from 0 to 1 where 1 was the highest score and 0 the lowest.

## Data analysis

Both bivariate and multivariate analysis was carried out to ascertain the relationship between the various factors and enrolment in the scheme. For the bivariate analysis cross-tabulation was carried out treating membership at the MHI scheme as dependent variable. Chi square test for categorical variable and t-test for continuous variable were used to test significance of each of the relationships. For the multivariate analysis, logistic regression models were used taking membership to the scheme as dependent variables. Stata/SE 14.1 has been used for analysis.

## Results

Among the 1956 households interviewed, 50.4% were member households and 49.6% were non members. Table 2 presents selected descriptive statistics of sample households and its members. A comparative picture between the member and the non member households is also presented.

**Table 2.** Factors influencing enrolment in Micro-health Insurance (MHI) scheme, Chakaria, Bangladesh.

Variables	Total sample	Enrolees	Non-enrolees	Differences
	N=1956	N=972	N=984	
Religion				
Islam	1,781	49.9	50.1	χ^2^ =1.556
other	175	54.9	45.1	*P* = 0.212
Marital status of main income earner of household				
Never married	254	51.2	48.8	χ^2^ = 0.0791
Ever married	1702	50.2	49.8	*P* =0.778
Sex of main income earner of household				
Male	1821	50.6	49.4	χ^2^ = 0.527
Female	135	47.2	52.8	*P* =0.468
Age of main income earner of household				
<30 years	441	50.3	49.7	χ^2^ = 0.0001
>=30 years	1515	50.4	49.6	*P* = 0.993
Education level of main income earner of household				
None	573	46.6	53.4	χ^2^ = 20.985
1-5 years	708	47.5	52.5	*P* <0.001
6-10 years	496	53.6	46.4	
10+ years	179	64.8	35.2	
Occupation of main income earner of household				
Day-labour	717	50.9	49.1	χ^2^ =34.739
Farmer	318	44.0	56.0	*P* <0.001
Private/public service holder	231	63.7	36.3	
Businessman	445	53.1	46.9	
Others	241	39.0	61.0	
Level of financial literacy of main income earner of household				
<1	1033	45.8	54.2	χ^2^ =18.279
=1	923	55.5	44.5	P=0.001
Household size category				
1-3	239	40.6	59.4	χ^2^ =17.367
4-6	1199	49.6	50.4	P<0.001
7+	518	56.6	43.4	
Average household size		5.8(SD±2.13)	5.4 (SD±2.01)	P=0.000
Household in villages with VHP				
Yes	282	67.7	32.3	χ^2^ =39.781
No	1,674	47.4	52.6	*P* <0.001
NGO membership of households				
Yes	912	53.2	46.8	χ^2^ = 5.443
No	1,044	47.9	52.1	*P* = 0.020
Chronic disease of any household member				
Yes	746	57.9	42.1	χ^2^ = 27.504
No	1,210	45.7	54.3	*P* = 0.000
Children aged below 5 years in household				
Yes	854	52.3	47.7	χ^2^ = 2.386
No	1,102	48.8	51.2	*P* = 0.122
40+ aged person				
Yes	1461	52.1	47.9	χ^2^ = 12.71
No	495	42.6	57.4	P=0.000
Asset index				
Lowest	392	50.0	50.0	χ^2^ = 10.445
Second	407	51.4	48.6	*P* = 0.033
Middle	393	44.8	55.2	
Fourth	376	49.5	50.5	
Highest	388	56.2	43.8	

## Demographic and health factors

Among the demographic factors, no significant difference was observed between enrolees and the non-enrolees in terms of age, sex and marital status of main earner, existence of children aged below five years in household and religion of household. However member households were found to have significantly larger household size (in terms of total number of household members) than the non members (5.84, SD± 2.13 vs. 5.36, SD± 2.01). Membership was higher among households with at least one member aged 40 years or above compared to those who had no member aged 40 years or above (52.1% vs. 42.6%). In terms of health status, existence of members with chronic illness in the household showed significant positive relation with membership status (Table 2).

## Socio-cultural and economic factors

The proportion of main earners with more than 10 years of schooling was found to be higher for the member households compared to the non-members (64.8% vs. 35.2%). Exploring the occupation category of the main earner of the households it was found that 64% of the main income earners of member households were mostly engaged in private/public service followed by business (53.1%), menial labour (50.9%), and farming (44.0%). Whereas the proportion of main earners engaged in private/public service was much lower amongst the non-enrolees (36%). A higher proportion of households from highest socioeconomic quintile were enrolled into the scheme compared to those who were not (56.2% vs. 43.8%). Enrolment was higher for households with main earners scoring full (55.5%) on financial literacy index (FLI = 1) compared to those who scored less than 1 in FLI (44.5%) (Table 2).

## Structural factors

Enrolment rates were higher among the households having NGO membership compared to those who did not have any NGO membership (53.2% vs. 46.8%). Households being located within the villages where the VHPs were situated had positive effect on enrolment. Around 68% of the households within the VHP villages enrolled as opposed to 47% of the households enrolling from villages that did not have VHPs (Table 2).

## Multivariate analysis

Crude and adjusted odds ratios are presented in Table 3. Socioeconomic status of household, which was a significant predictor of membership in bivariate analysis, became non-significant after controlling for other variables.

**Table 3.** Unadjusted and adjusted association between household’s decision context and enrolment.

	Crude		Adjusted	
Variables	OR (95% CI)	P-value	AOR (95 % CI)	P-value
Main earner education		< 0.001		0.04
Illiterate	1.0		1.0	
1–5 years of schooling	1.0 (0.8, 1.3)		1.1 (0.9, 1.4)	
6–10 years of schooling	1.3 (1.1, 1.7)		1.3 (1.0, 1.8)	
10+ years of schooling	2.1 (1.5, 3.0)		1.9 (1.2, 2.9)	
Main earner occupation		< 0.001		0.002
Farmer	1.0		1.0	
Day-labour	1.3 (1.0, 1.7)		1.3 (1.0, 1.8)	
Public/private service holder	2.2 (1.6, 3.2)		1.6 (1.1, 2.4)	
Businessman	1.5 (1.1, 1.9)		1.2 (0.9, 1.6)	
Others	0.8 (0.6, 1.1)		0.8 (0.5, 1.1)	
Main earner financial literacy index		< 0.001		0.000
< 1	1.0		1.0	
= 1	1.5 (1.2, 1.8)		1.5 (1.2, 1.8)	
Household size		< 0.001		0.013
1–3	1.0		1.0	
4–6	1.4 (1.1, 1.9)		1.3 (1.0, 1.8)	
7+	1.9 (1.4, 2.6)		1.6 (1.2, 2.3)	
Household within VHP village		< 0.001		
No	1.0		1.0	0.000
Yes	2.3 (1.8, 3.0)		2.1 (1.6, 2.7)	
NGO membership		0.002		
No	1.0		1.0	0.04
Yes	1.2 (1.0, 1.5)		1.2 (1.0, 1.5)	
Chronic disease of any member		< 0.001		0.000
No	1.0		1.0	
Yes	1.6 (1.4, 2.0)		1.5 (1.2, 1.8)	
At least one member aged 40 or above				
No	1.0	0.000	1.0	0.035
Yes	1.5 (1.2, 1.8)		1.3 (1.0, 1.6)	
Asset index		0.033		0.138
Lowest	1.0		1.0	
Second	1.1 (0.8, 1.4)		1.0 (0.7, 1.3)	
Middle	0.8 (0.6, 1.1)		0.7 (0.5, 0.9)	
Fourth	1.0 (0.7, 1.3)		0.8 (0.6, 1.1)	
Highest	1.3 (1.0, 1.7)		0.8 (0.6, 1.2)	

OR = Odds ratio; AOR = Adjusted odds ratio; CI = Confidence Interval

Education was still found to be an important predictor of enrolment. Households with main income earner having 10+ years schooling were more likely to enrol (AOR 1.9) as compared to households with illiterate main earner. Households with main earners who scored full in financial literacy index (FLI = 1) were more likely to enrol (AOR 1.5; p = 0.000) in relation to those with main earners not scoring full (FLI< 1).

As the number of members suffering from chronic illness increased in a household they were more likely to join the scheme. Odds of households with members suffering from chronic illness were 1.5. Further, households that had at least one member aged 40 or above were more likely to enrol compared to those that did not have any member aged 40 or above (AOR 1.3).

In terms of occupation, households where occupation of main earner was public/private service had the highest odds ratio of 1.6 (reference category ‘farmers’).

NGO membership had positive influence on enrolment decision. The odds of membership for households with at least one member having NGO membership were higher than those who had none (AOR 1.2).

Distance to the health centres played an important role in people’s decision to enrol into the MHI scheme. Households who lived in the villages where the village health posts (VHPs) were situated were more likely to join the scheme than those living further away from the VHPs. The odds of households within the VHP villages joining the scheme were 2.1 compared to those not living in VHP villages.

## Discussion

The current paper seeks to understand factors that influence the decision to enrol in micro health insurance (MHI) schemes in a low-income country like Bangladesh. The Outreville’s insurance demand framework identified significant factors that were multidimensional in nature covering economic, socio-cultural, demographic and structural context of the respondents. Factors that had significant influence on decision to enrol included: education, financial literacy and occupation of the main income earner of a household, the size of the household in terms of total members, distance of household from health facilities, membership in any NGO and households with a member suffering from a chronic illness.

Enrolment was not equitable across all groups of people. Households with an educated main earner were more likely to join MHI schemes compared to those where the main earner was illiterate. The influence of education on enrolment in MHI schemes has been documented elsewhere [25–29]. This could be due to better understanding of the concept of health insurance among the educated group. Education has far reaching implications in people’s lives. Along with education comes access to information on myriad issues, which in turn prompts informed decision-making. Similarly financial literacy encouraged membership. The low rate of participation among households with low level of education may also point to the lacking in the marketing strategy of the scheme that led to more educated households joining. To ensure participation from households with low-literacy level, the scheme would need to invest on designing marketing techniques that are more user-friendly for clients belonging to lower literacy group.

Enrolment probability was higher among households who were more likely to use or were in greater need of healthcare services. The findings show that households that had at least one member with chronic illness were more likely to join the program. From the user point of view, micro health insurance is providing effective coverage in this case. However, from the provider point of view, this is a case of classical insurance market failure through moral hazard and adverse selection. Experience of moral hazard and adverse selection has been documented in earlier studies [30]. Repeated unnecessary use and higher risk group disproportionately joining the scheme would eventually threaten survival of the scheme. A few remedies such as, excluding some pre-conditions, introducing waiting time, co-payment, mandatory enrolment [26], group enrolment, and sliding scale premium have been tried in different parts of the world in minimizing the financial risk resulting from moral hazard and adverse selection [31,32]. Each of these methods has their benefits and limitations. Introducing a mandatory scheme enrolment in rural Bangladesh and other similar settings where the majority of the population is involved in informal economy is difficult. Group insurance, as tested in China and Ghana, can be one of the choices making enrolment mandatory for a specific group of people. However, group insurance can also run into the problem of attracting high risk groups. Despite the fact that excluding pre-existing conditions can potentially limit the impact of such schemes in terms of providing financial risk protection for poor household, it might be required towards the beginning while the scheme awaits financial stability.

Type of occupations was another major demand influencing factor. Households engaged in occupations that had a constant flow of income were more likely to join the scheme. As a result, service holders were more likely to join compared to those engaged in menial labour. Private or public service holders have a comparatively higher socioeconomic status and they work for a fixed salary which could be the reason encouraging those households to join MHI schemes. On the contrary, clients engaged in agricultural work and other such occupation had seasonal income and thus their ability to pay fluctuated with income flow. Matching premium collection time with the income flow might encourage more of this group of people to join.

The effect of asset quintile on enrolment was found to be insignificant and almost equal proportions of people from the different socioeconomic strata joined the scheme. This could be a result of the safety-net offered by the special outpatient package where the premium of the households from lower socioeconomic status was subsidized. The introduction of the special outpatient package was thus an equity enhancing measure of the *Amader Shasthya* scheme. Literature so far has identified setting of an appropriate level of premium as a major challenge for the MHI schemes in the developing countries [33]. MHI schemes around the developing world have been found to charge a flat rate premium for its clients. This is primarily due to the fact that the client base for MHI schemes mostly consists of the informal sector and charging a sliding scale premium according to people’s level of income is complex. However, a flat rate premium tends to make the system highly regressive as poor people in this system contribute a higher proportion of their income than wealthier people [34–36]. Safety-nets where premium for those with lower ability-to-pay are subsidized or exempted have the potential to bring in the desired level of equity [34].

Membership in development programmes offered by NGOs, particularly the micro finance programmes, was found to have significant positive influence on decision to enrol. These income generating programmes give the households access to additional funds that they can use to pay for the premium. Additionally involvement with development programmes also gives this group exposure to knowledge and information, which the other group does not have access to or are simply not aware of. The higher likelihood of NGO members joining the Chakaria health card scheme might also be indicative of the in-built solidarity that the members of such development programs share. Earlier studies have also found the success of MHI schemes to be dependent on the level of social capital [2,37,38]. MHI schemes can tap into the solidarity among the different groups of people. The dominant religion in Bangladesh is Islam which mandates the better-off Muslims to donate a proportion of their income (known as *Zakat*) when their savings exceeds a certain level and it is used as a poverty alleviation tool [39]. MHI schemes can also think of accessing the zakat funds to subsidize premiums for the poor. MHI schemes can bank on the groups of people identified in this study who are in favour of participating in MHI schemes and use them to influence their peer and neighbours.

Distance between households and the health centre through which the MHI scheme provided health services played a significant role in the decision to enrol. Villagers living in places far from the health centres were less likely to join the MHI scheme compared to those who lived nearby. The significant impact of non financial barriers has resulted in varying effects of MHI schemes in different parts of developing countries [40–45]. In designing MHI schemes for rural Bangladesh, the provider thus has to acknowledge these non financial barriers and work towards narrowing the inequities resulting from them.

The findings from this study identify particular groups of people who were more inclined towards joining a health insurance scheme. Further studies need to be carried out among these people to understand the factors that attract people to join. At the same time, the behaviour of those who did not join also needs to be studied carefully to identify factors that may deter potential clients from joining any insurance schemes.

Limitation of the study: The list of determinants of enrolment tested in the current paper is not an exhaustive one. A few major determinants of insurance enrolment like risk attitude of members, trust on provider organization and availability of alternate insurance or other health service options have not been considered in the analysis due to absence of data on these. Further to this, the MHI scheme analysed in this paper was operated by a very reputable and trusted organization in Bangladesh (icddr,b). Enrolment in MHI scheme has shown to be influenced by trust to a great extent [6,10] and therefore the findings presented here may have been influenced by the reputation of icddr,b and the analysis could not separate out this effect. This might limit the generalization of the findings to some extent.

## Conclusion

It is needless to say that micro health insurance is not a silver bullet in increasing efficiency of health financing mechanisms and ensuring access to quality healthcare for all in a low-resource setting like Bangladesh. However, the findings of this study are expected to have significant implications in terms of designing similar health insurance schemes, particularly in terms of designing demand-driven and context-adapted schemes that have greater potential to attract a larger client pool, ensure effective risk pooling and eventually expedite the achievement of universal health coverage. Micro health insurance has come under discussion in the policy arena of Bangladesh in recent times with its mention in the current national health financing strategy 2012–32 which aims to achieve universal health coverage. MHI has been identified as an intermediate step before initiating a social health insurance scheme that will allow the country to build its capacity to manage publicly funded large scale health insurance schemes. In this light of strategic thinking, the learning’s from the study can feed into the national health policy of Bangladesh to better equip it in designing an effective health financing tool.

## Paper context

Micro health insurance can protect the poor from healthcare related financial shocks. However, low uptake and high dropouts challenge its financial sustainability. Understanding the multidimensional factors influencing enrolment decision is important. The current study analyzes enrolment determinants in four dimensions of demand: Economic, social and cultural, demographic, and structural. The findings are expected to have significant implications in terms of designing similar health insurance schemes, particularly in terms of designing demand-driven and context adapted schemes that have greater potential to attract a larger client pool, ensure effective risk pooling and eventually expedite the achievement of universal health coverage.

## References

[1] Preker AS, Carrin G, Dror D, et al Effectiveness of community health financing in meeting the cost of illness. Bull World Health Organ. 2002;80: 143–10. Epub 2002/04/16.PubMed PMID: 11953793; PubMed Central PMCID: PMCPmc2567719. 11953793 PMC2567719

[2] Donfouet HPP, Mahieu P-A. Community-based health insurance and social capital: a review. Health Econ Rev. 2012;2:5 PubMed PMID: PMC3402932. 22828204 10.1186/2191-1991-2-5 PMC3402932

[3] Develtere P, Doyen G, Fonteneau B, editors. Micro-insurance and health care in developing countries: an international picture. Belgium: Hilde Talloen; 2004.

[4] Matin I, Imam N, Ahmed SM Micro Health Insurance (MHI) pilot of BRAC: a demand side study. Dhaka: BRAC; 2005.

[5] Werner WJ Micro-insurance in Bangladesh: risk protection for the poor? J Health Popul Nutr. 2009;27:563–573. 19761089 10.3329/jhpn.v27i4.3402 PMC2928102

[6] Iqbal M, Chowdhury AH, Mahmood SS, Mia MN, Hanifi SMA, Bhuiya A. Socioeconomic and programmatic determinants of renewal of membership in a voluntary micro health insurance scheme: evidence from Chakaria, Bangladesh. Global Health Action 2017;10(1):1287398. 10.1080/16549716.2017.1287398 PMC5496168 28471332

[7] Biener C, Eling M Insurability in microinsurance markets: an analysis of problems and potential solutions. The Geneva Papers. 2012;37:77–107.

[8] Révész P Chapter 1 - Definitions and generalities In: Révész P, editor. The laws of large numbers. New York and London: Academic Press; 1967 p. 31–38.

[9] Churchill C, editor. Protecting the poor: a microinsurance compendium. Germany: ILO; 2006.

[10] Dror DM, Hossain SAS, Majumdar A, et al What factors affect voluntary uptake of community-based health insurance schemes in low- and middle-income countries? A systematic review and meta-analysis. PLoS One. 2016;11:e0160479. 27579731 10.1371/journal.pone.0160479 PMC5006971

[11] Macha J, Kuwawenaruwa A, Makawia S, et al Determinants of community health fund membership in Tanzania: a mixed methods analysis. BMC Health Serv Res. 2014;14:538 PubMed PMID: PMC4246628. 25411021 10.1186/s12913-014-0538-9 PMC4246628

[12] Adebayo EF, Ataguba JE, Uthman OA, et al Factors that affect the uptake of community-based health insurance in low-income and middle-income countries: a systematic protocol. BMJ open. 2014;4:2. 10.1136/bmjopen-2013-004167 PMC3927816 24531450

[13] Hamid SA, Roberts J, Mosley P Can micro health insurance reduce poverty? Evidence from Bangladesh. J Risk Insur. 2011;78:57–82.

[14] Ahmed MU, Islam SK, Quashem MA, et al Health microinsurance: a comparative study of three examples in Bangladesh. CGAP working group on microinsurance. Good and Bad Practices. Case Study No. 13. Germany: ILO; 2005.

[15] Outreville J The relationship between insurance and economic development: 85 empirical papers for a review of the literature. Risk Manag Insurance Rev. 2013;16:71–122.

[16] Hanifi MA, Sultana A, Mia MN, et al Chakaria health and demographic surveillance system report-2016. Focusing on the sustainable development goals. Dhaka: icddr,b, 2017 (Contract No.: Scientific Report no. 137).

[17] Ministry of Health and Family Welfare (MOHFW). Health Bulletin 2015: Chakaria Upazila Health Complex Management Information System (MIS), Directorate General of Health Services (DGHS), Ministry of Health & Family Welfare (MOHFW), 2015.

[18] Van Doorslaer E, O’Donnell O, Rannan-Eliya RP, et al Effect of payments for health care on poverty estimates in 11 countries in Asia: an analysis of household survey data. The Lancet. 2006;368:1357–1364. 10.1016/S0140-6736(06)69560-3 17046468

[19] Advocate Sultana Kamal DI, Dr. Sumaiya K, Mohammad Rafiqul H Governance challenges in the health sector and the way out. Dhaka: Transparency International Bangladesh; 2014.

[20] Icddr B Recent learnings from a community health insurance scheme in Chakaria. Bangladesh Dhaka: ICDDR,B; 2013 (Contract No.: 1.).

[21] Iqbal M, Chowdhury AH, Ss M, et al Socioeconomic and programmatic determinants of renewal of membership in a voluntary micro health insurance scheme: evidence from Chakaria, Bangladesh. Global Health Action Epub 2017/ 05/05 2017;10:1287398 PubMed PMID: 28471332; PubMed Central PMCID: PMCPMC5496168. 28471332 10.1080/16549716.2017.1287398 PMC5496168

[22] Vyas S, Kumaranayake L Constructing socio-economic status indices: how to use principal components analysis. Health Policy Plan. 2006;21:459–468. 17030551 10.1093/heapol/czl029

[23] Howe LD, Hargreaves JR, Huttly SR Issues in the construction of wealth indices for the measurement of socio-economic position in low-income countries. Emerg Themes Epidemiol. 2008;5:1. 18234082 10.1186/1742-7622-5-3 PMC2248177

[24] Filmer D, Pritchett LH Estimating wealth effects without expenditure data—or tears: an application to educational enrollments in states of India. Demography. 2001;38:115–132. 11227840 10.1353/dem.2001.0003

[25] Kimani JK, Ettarh R, Warren C, et al Determinants of health insurance ownership among women in Kenya: evidence from the 2008-09 Kenya demographic and health survey. Int J Equity Health. 2014;13:27 PubMed PMID: 24678655; PubMed Central PMCID: PMC3973618. 24678655 10.1186/1475-9276-13-27 PMC3973618

[26] Blanchet NJ, Fink G, Osei-Akoto I The effect of Ghana’s National Health Insurance Scheme on health care utilisation. Ghana Med J. 2012;46: 76–84. Epub 2012/09/04.PubMed PMID: 22942455; PubMed Central PMCID: PMCPmc3426378. 22942455 PMC3426378

[27] Chankova S, Sulzbach S, Diop F Impact of mutual health organizations: evidence from West Africa Health Policy Plan. 2008;23(4):264–276. 10.1093/heapol/czn011 PMC2430720 18480126

[28] Lammers J, Warmerdam S Adverse selection in voluntary micro health insurance in Nigeria. Amsterdam: Amsterdam Institute for International Development; 2010.

[29] Wang H, Yip W, Zhang L, et al Community-based health insurance in poor rural China: the distribution of net benefits. Health Policy Plan. 2005;20:366–374. 16143589 10.1093/heapol/czi045

[30] Parmar D, Souares A, de Allegri M, et al Adverse selection in a community-based health insurance scheme in rural Africa: implications for introducing targeted subsidies. BMC Health Serv Res. 2012;12:181. 22741549 10.1186/1472-6963-12-181 PMC3457900

[31] Wang H, Zhang L, Yip W, et al Adverse selection in a voluntary Rural Mutual Health Care health insurance scheme in China. Soc Sci Med Epub 2006/ 04/26 2006;63:1236–1245. PubMed PMID: 16635541. 16635541 10.1016/j.socscimed.2006.03.008

[32] Ahmed S, Sarker AR, Sultana M, et al Adverse selection in community based health insurance among informal workers in Bangladesh: an EQ-5D Assessment. Int J Environ Res Public Health. 2018;15:242 PubMed PMID: PMC5858311. 10.3390/ijerph15020242 PMC5858311 29385072

[33] Udeh EI, Onwujekwe OE, Adewole DA, et al Exploring the threshold premium for viable community based health insurance schemes in Nigeria. BMC Res Notes. 2016;9:383 PubMed PMID: PMC4971742. 27485606 10.1186/s13104-016-2185-1 PMC4971742

[34] OXFAM Universal health coverage: why health insurance schemes are leaving the poor behind. Oxford, UK: OXFAM; 2013 p. 176.

[35] Mills A, Ataguba JE, Akazili J, et al Equity in financing and use of health care in Ghana, South Africa, and Tanzania: implications for paths to universal coverage. The Lancet. 2012;380:126–133. 10.1016/S0140-6736(12)60357-2 22591542

[36] McIntyre D Health service financing for universal coverage in east and southern Africa. Harare: EQUINET; 2012 p. 95.

[37] Carrin G Social health insurance in developing countries: A continuing challenge. Int Soc Secur Rev. 2002;55:2.

[38] Dror I Social capital and microinsurance - Insights from field evidence in India. Microfinance Insights. 2007:22.

[39] Kaleem A, Ahmed S The quran and poverty alleviation: a theoretical model for charity-based Islamic microfinance institutions. Nonprofit Voluntary Sector Quarterly. 2009 DOI:10.1177/0899764009332466

[40] Asenso-Okyere WK, Osei-Akoto I, Anum A, et al Willingness to pay for health insurance in a developing economy. A Pilot Study of the Informal Sector of Ghana Using Contingent Valuation. Health Policy. 1997;42:223–237. 10176302 10.1016/s0168-8510(97)00069-9

[41] Asgary A, Willis K, Taghvaei AA, et al Estimating rural households’ willingness to pay for health insurance. Eur J Health Econ. 2004;5:209–215. 15714341 10.1007/s10198-004-0233-6

[42] Garrett L, Chowdhury AMR, Pablos-Méndez A All for universal health coverage. The Lancet. 2009;374: 1294–1299. PubMed PMID: 199052939; 19698983. 10.1016/S0140-6736(09)61503-8 19698983

[43] Basinga P, Gertler PJ, Binagwaho A, et al. Paying primary health care centers for performance in Rwanda. Washington, DC: The World Bank, 2010 Policy Research Working Paper No. 5190.

[44] Toonen J, Canavan A, Vergeer P, et al. Learning lessons on implementing performance based financing from a multi-country evaluation. Amsterdam, Royal Tropical Institute: Royal Tropical Institute in collaboration with Cordaid and the World Health Organization, 2009.

[45] Basaza R, Criel B. Van der Stuyft P Community health insurance in Uganda: why does enrolment remain low? A view from beneath. Health Policy. 2008;87:172–184. 18280608 10.1016/j.healthpol.2007.12.008

